# Hints for Avoiding Rheumatism

**Published:** 1885-06-20

**Authors:** 


**Hints for Avoiding Rheumatism.**—Persons with rheumatic tendencies should carefully avoid living in damp houses or on damp sites. There is always more rheumatism in valleys than on plains and heights. Avoid getting wet, wear flannel next the skin, and drink no beer. Beer and wine, and excess of animal food always tend to encourage rheumatic complaints. The diet should consist largely of vegetable food.—*The Laws of Life.*

